# Advanced deep learning models for phenotypic trait extraction and cultivar classification in lychee using photon-counting micro-CT imaging

**DOI:** 10.3389/fpls.2024.1358360

**Published:** 2024-02-29

**Authors:** Mengjia Xue, Siyi Huang, Wenting Xu, Tianwu Xie

**Affiliations:** ^1^ Institute of Radiation Medicine, Fudan University, Shanghai, China; ^2^ Britton Chance Center for Biomedical Photonics, Wuhan National Laboratory for Optoelectronics, Huazhong University of Science and Technology, Wuhan, Hubei, China

**Keywords:** plant phenomics, micro-CT, lychee phenotypic traits, deep learning, non-destructive

## Abstract

**Introduction:**

In contemporary agronomic research, the focus has increasingly shifted towards non-destructive imaging and precise phenotypic characterization. A photon-counting micro-CT system has been developed, which is capable of imaging lychee fruit at the micrometer level and capturing a full energy spectrum, thanks to its advanced photon-counting detectors.

**Methods:**

For automatic measurement of phenotypic traits, seven CNN-based deep learning models including AttentionUNet, DeeplabV3+, SegNet, TransUNet, UNet, UNet++, and UNet3+ were developed. Machine learning techniques tailored for small-sample training were employed to identify key characteristics of various lychee species.

**Results:**

These models demonstrate outstanding performance with Dice, Recall, and Precision indices predominantly ranging between 0.90 and 0.99. The Mean Intersection over Union (MIoU) consistently falls between 0.88 and 0.98. This approach served both as a feature selection process and a means of classification, significantly enhancing the study's ability to discern and categorize distinct lychee varieties.

**Discussion:**

This research not only contributes to the advancement of non-destructive plant analysis but also opens new avenues for exploring the intricate phenotypic variations within plant species.

## 1 Introduction

Lychee (Litchi chinensis Sonn.) is a subtropical fruit enjoyed for its unique taste and nutritional benefits, primarily grown in parts of Asia (Menzel, 1985). Recent research highlights lychee’s potential health properties, including fighting cancer and bacteria, as well as its antioxidant capabilities (Zhang et al., 2021). As interest grows in how genes affect lychee traits, some studies are uncovering errors in cultivar naming (Liu and Mei, 2005) and using new genetic markers for plant diversity studies (Tran et al., 2019).

New technologies like micro-CT scans are transforming how we study plants, allowing us to see the details of plant structures without destroying them (Keklikoglou et al., 2021). This method is proving useful for examining plant parts like roots in their natural soil environment (Kurogane et al., 2021) and for studying flower shapes more efficiently (Begot et al., 2022). Yet, to fully understand plants, further interests need to move from looking at images to measuring them accurately (Karahara et al., 2023).

Deep learning has been utilized to measure plant traits for better crop breeding (Wu et al., 2021), assisting in harvesting fruits like lychee (Xie et al., 2022), and even works on portable devices in the field (Jiao et al., 2022). Researchers have also made it easier to tell different lychee cultivars apart just by their shape (Osako et al., 2020). Not only can it help identifying plants from complex environment, but also can rebuilt 3D models and get phenotypic traits when analyzing CT images of the plant. A nondestructive method for more accurate and efficient automatic acquisition of comprehensive phenotypic traits has been developed in the passion fruit (Lu et al., 2023) and the coconut (Yu et al., 2022).

In this study, a state-of-the-art photon-counting micro-CT technology was used to investigate the internal structure of lychees, which is the first attempt as far as we know. Then seven CNN-based deep learning models were utilized to segment the CT images and extract phenotypic traits automatically. We compared the algorithm-driven approach with manual analysis to see whether the approach well worked. To examine which phenotypic traits are the key characteristics of a lychee, machine learning based on small-sample training is used for feature selection and classification.

## 2 Methods

### 2.1 Materials

**Figure 1:**
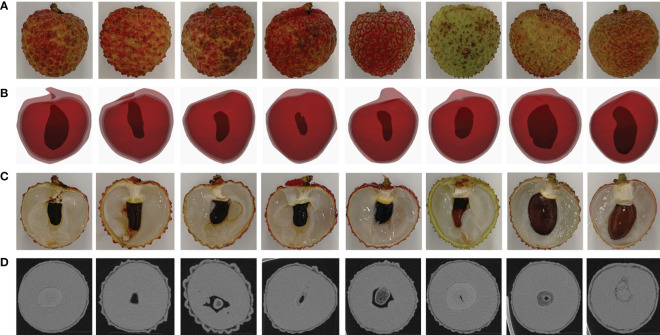
Four morphologically representative lychee samples. **(A)** RGB images of lychees. **(B)** 3D NURBS model of lychee constructed from Micro-CT. **(C)** Cross-sectional view of lychee. **(D)** Tomogram images of lychee cross-section from Micro-CT.

In this study, 80 lychees (10 individuals per species) were randomly selected from eight distinct species for photon-counting micro-CT imaging and manual measurements. The species sampled were carefully chosen to represent a wide range of lychee varieties cultivated in China. These included: 1. ‘Xian Jin Feng’ from Guangzhou, Guangdong; 2. ‘Gui Wei’ from Zhanjiang, Guangdong; 3. ‘Jing Gang Hong Nuo’ from Guangzhou, Guangdong; 4. ‘Nuo Mi Ci’ from Guangdong; 5. ‘Fei Zi Xiao’ from Guangdong; 6. ‘Yu He Bao’ from Yangjiang, Guangdong, noted for its larger seed size; 7. ‘Ji Zui’ from Zhanjiang, Guangdong; 8. ‘Hei Ye’ from Fujian. The samples were meticulously collected to ensure they accurately represented each variety’s unique traits. **Figure 1** presents eight different species of lychee, each row showing a unique type through real, modeled, and cross-sectional images.

### 2.2 Photon-counting micro-CT imaging

**Figure 2:**
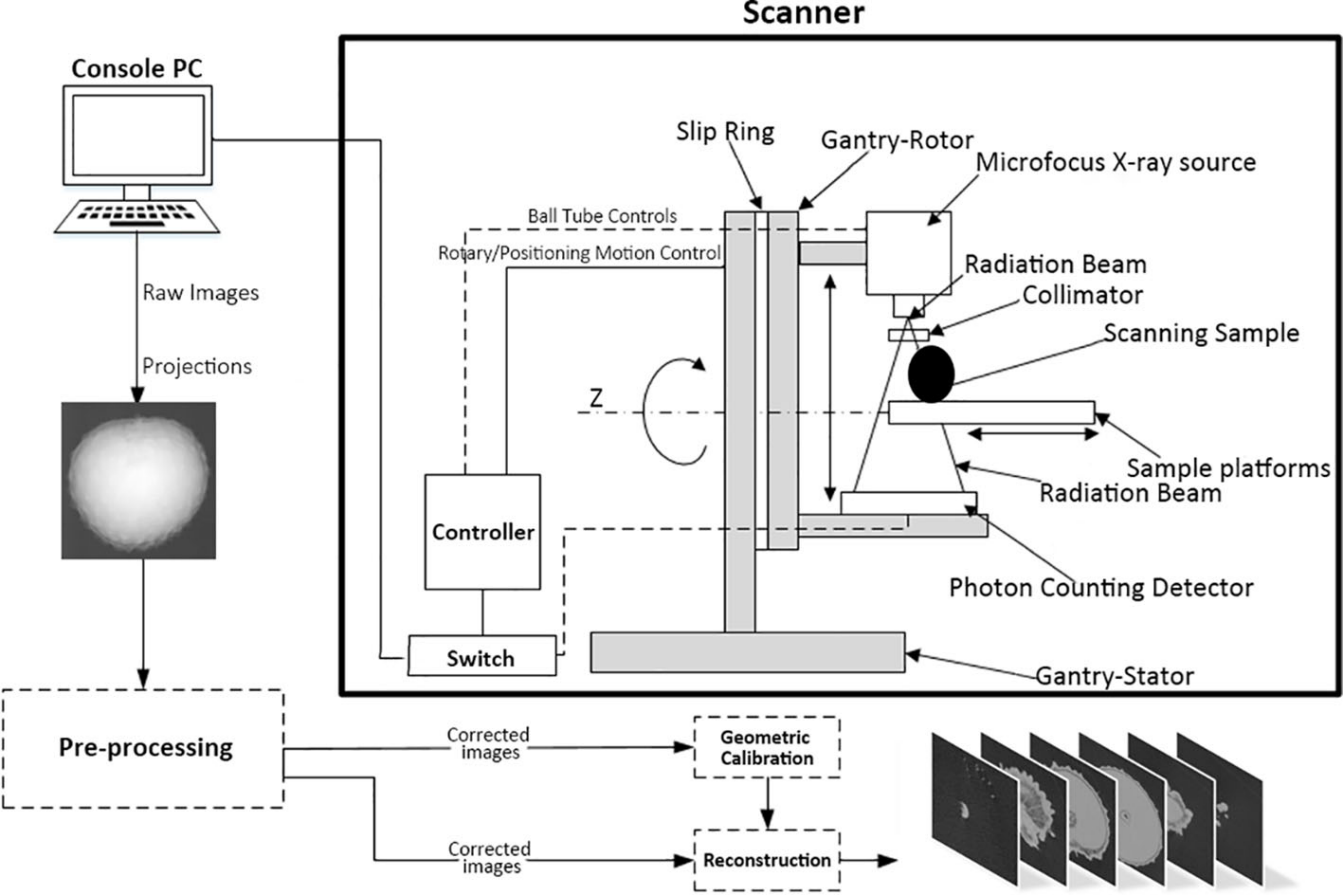
Photon-counting micro-CT scanner design. By rotational scanning the lychee samples, the MFX and the photon-counting detectors transmit data to the Console PC, which then generates CT images through pre-processing and reconstruction steps.

To our knowledge, it represents the first instance of such advanced technology being utilized in agricultural field. The research employed a state-of-the-art photon-counting micro-computed tomography system to acquire images, engineered for acquiring high-resolution images with a voxel resolution finer than 100 micrometers, as depicted in **Figure 2**. This imaging system is able to reveal intricate details that are not discernible through traditional CT imaging. The lychee samples are placed on the sample platforms situated between the Microfocus X-ray source (MFX) and the photon-counting detectors. During the scanning process, both the MFX and the detectors rotate around the sample. This movement is crucial as it enables the system to capture a multitude of projection images from various angles, effectively ‘slicing’ through the sample in a non-destructive manner. This advanced imaging technique provided us with detailed crosssectional images of the lychee fruit, enabling precise measurements and analysis of internal structures that are otherwise difficult to assess with traditional methods. It is not only allowed us to visualize the internal architecture of the lychee fruits, but also to construct three-dimensional models for a more comprehensive evaluation of their physical attributes.

### 2.3 Segmentation models

The research utilized seven CNN-based deep learning models, including AttentionUNet (Mishra et al., 2018), DeeplabV3+ (da Cruz et al., 2022), SegNet (Alqazzaz et al., 2019), TransUNet (Chen et al., 2021), UNet (Ronneberger et al., 2015), UNet++ (Zhou et al., 2019), and UNet3+ (Huang et al., 2020), to segment CT images and effectively identified the kernel, pulp, endocarp and epicarp parts of a lychee. Accurate segmentation by these models is fundamental for analyzing the lychee’s internal features, which is essential for understanding its physical traits.

The UNet model is a primary tool in our study due to its efficient design that captures important features in the images and then reconstructs a segmented image that includes all necessary details. SegNet enhances this process by using a special technique that improves the model’s ability to capture and recreate finer details. DeeplabV3+ goes a step further by processing different image sizes effectively, which is important when dealing with various parts of the lychee. Attention UNet, UNet++, and UNet3+ build on this by focusing on the most important features in the image and combining information at different scales to create a more accurate picture. TransUNet stands out by blending the strengths of two architectures to provide a balance between a broad view and detailed aspects of the images.

The study processed a total of 6480 images, about 810 images for each lychee species. The dataset was then randomly divided, with 80% allocated for training models and the remaining 20% reserved for validation purposes. To define the performance of each CNN-based model in comparison to ground truth segmentations, the following four quantitative indicators were used: mean intersection over union (mIOU), dice similarity coefficient (DSC), precision, and recall.

### 2.4 Trait extraction approach

Following the delineation of the lychee’s kernel, pulp, endocarp, and epicarp, morphological characteristics of the fruit were meticulously extracted. Key morphological traits examined in this study are concisely summarized in a dedicated table. Quantitative data for these traits have been rigorously compiled, as evident in the ‘Values’ column of **Table 1**. The measurements were initially obtained manually to establish a baseline for subsequent comparison.

**Table 1:** Extraction of morphological traits from lychee CT images.

Traits Class	Morphological Traits		Values		Unit
	Description	Abbreviation	Mean ± SD	Range	
Lychee Fruit Traits	Fruit Weight	*W_Fruit*	24.40 ± 3.76	18.43 ~ 35.13	g
	Fruit Length	*L_Fruit*	37.51 ± 2.81	31.54 ~ 46.64	mm
	Fruit Width	*D_Fruit*	35.12 ± 2.24	30.25 ~ 40.64	mm
	Fruit Height	*H_Fruit*	36.03 ± 2.35	32.29 ~ 46.51	mm
Kernel Traits	Kernel Weight	*W_Kernel*	1.17 ± 0.93	0.24 ~ 3.65	g
	Kernel Length	*L_Kernel*	10.89 ± 2.03	6.50 ~ 16.22	mm
	Kernel Width	*D_Kernel*	8.94 ± 2.56	5.21 ~ 13.35	mm
	Kernel Height	*H_Kernel*	17.49 ± 3.02	12.00 ~ 24.43	mm
Pulps Traits	Pulps Weight	*W_Pulps*	4.84 ± 0.95	2.72 ~ 7.96	g
	Pulps Length	*L_Pulps*	31.24 ± 2.18	27.72 ~ 38.15	mm
	Pulps Width	*D_Pulps*	32.14 ± 2.37	27.43 ~ 39.43	mm
	Pulps Height	*H_Pulps*	32.46 ± 2.45	27.43 ~ 39.34	mm
Others	RGB Color - R	R	107 ± 13	84 ~ 142	–
	RGB Color - G	G	55 ± 13	33 ~ 100	–
	RGB Color - B	B	26 ± 4	17 ~ 35	–
	Surface Area	Area	534.11 ± 64.12	430.76 ~ 703.86	0.7 cm²

Concurrently, a series of algorithms was developed for the automated quantification of these traits, facilitating the validation and accuracy assessment against manual measurements. Pearson Correlation analysis, applied following data acquisition, illuminated the interrelationships among various traits. The use of Linear Discriminant Analysis (LDA) proved instrumental in determining the significance of each trait for distinguishing between lychee varieties.

In the final stage of our research, the study applied classification techniques including Support Vector Machine (SVM), Random Forest Classifier (RFC), and LDA for species categorization of lychee. To optimize the classification performance with a limited sample size, preprocessing strategies like Normalization, Stratified Cross-Validation (CV), and Leave-One-Out Cross-Validation (LOO CV) were integrated. These methods contributed significantly to the robustness and accuracy of the species classification model.

## 3 Results

### 3.1 Projection image obtained from photon-counting micro-CT system

**Figure 3:**
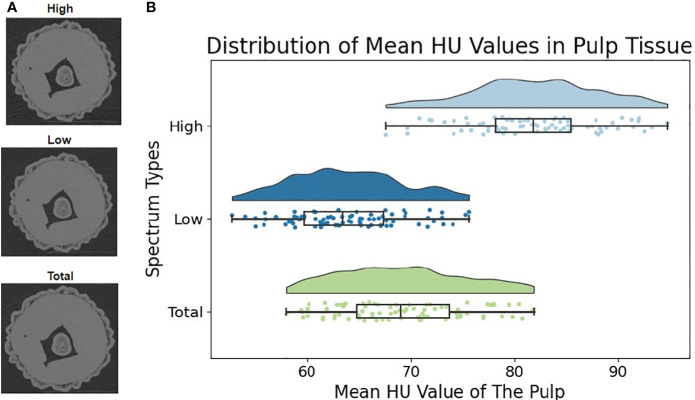
Multi-energy spectral analysis based on photon-counting detectors. **(A)** The left column features three sub-figures, each reconstructed using different energy spans. **(B)** The violin plot with a box plot on the right side were used to reveal the distribution of average Hounsfield Unit (HU) values in lychee pulp tissues.

Photon-counting detectors can calculate the number of photons at each different energy levels with highly energy resolutions. It will generate spectrums with full energy span, allowing for detailed information acquisition at both low and high energy levels. This tool is utilized in our photon-counting micro-CT setup to obtain clear and detailed images from CT scans. **Figure 3** illustrates the results achievable from this characteristic of the system. This graph is crucial for understanding the variations in density and composition of the samples at different energy levels.

### 3.2 Model performance in image segmentation

**Figure 4:**
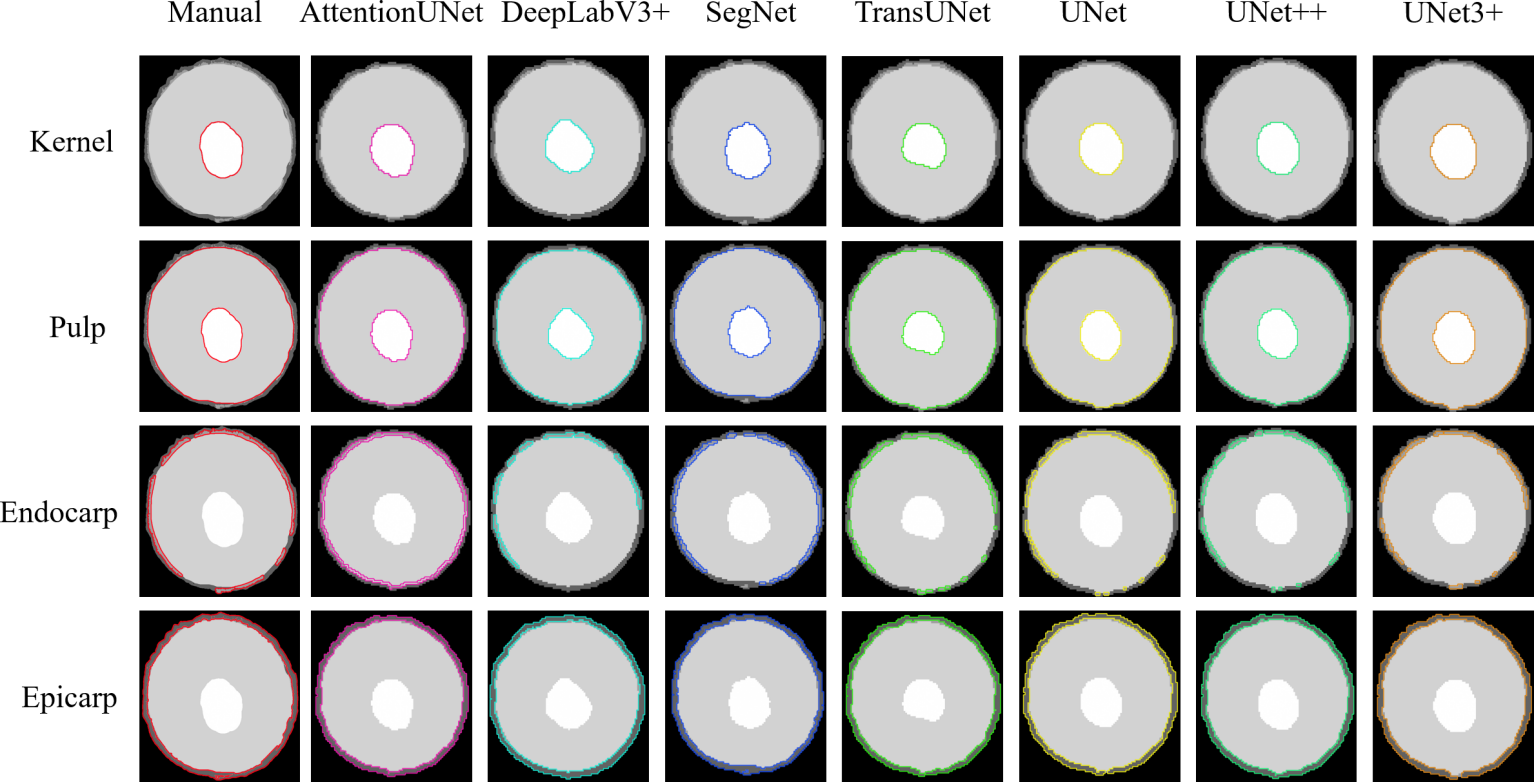
Segmentation results. The images presented in the figure are from the segmentation results of the same sample in the test set trained by different models. The first column acts as a reference, presenting the manual segmentation of the lychee fruit’s components, namely the kernel, pulp, endocarp, and epicarp. The following columns exhibit the segmentation outcomes from different models, including AttentionUNet, DeepLabV3+, among others. These segments are highlighted with color-coded outlines, facilitating a swift comparison of each model’s precision in relation to the manual segmentation.

From the segmentation results of each model on the test set, an image with same naming was selected for comparative analysis. The segmentation accuracy of models is visually represented in **Figure 4**. This manual segmentation is the standard we use to judge the automated models’ performance. The clear visual differences between the manual and automated segmentation provide us with insight into which model most closely replicates the expert’s accuracy, guiding us in choosing the best model for our analysis.

**Figure 5:**
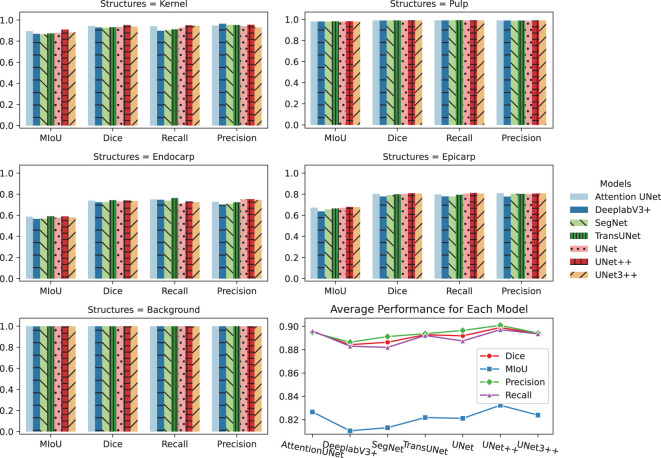
Performance comparison of deep learning segmentation models. The initial five subfigures display the performance of seven CNN-based models in terms of MIoU, Dice, Recall, and Precision indices, specifically when segmenting different parts of the lychees, including the Kernel, Pulp, Endocarp, Epicarp, and Background. The final sub-figure provides an aggregated view of the average performance for each model.

After the visual comparisons, **Figure 5** offers a detailed quantitative analysis of the performance of each deep learning model in segmenting various components of the lychee fruit. This analysis includes metrics such as the Mean Intersection over Union (MIoU), Dice coefficient, Recall, and Precision. These models exhibit remarkable classification capabilities, particularly for the Kernel, Pulp, and Background, with most evaluation indices approaching or exceeding 0.88, and in some cases, surpassing 0.98. They have an excellent Dice, Recall, and Precision index ranging mostly between 0.90 and 0.99, while the Mean Intersection over Union (MIoU) is recorded mostly between 0.88 and 0.98. UNet++ and TransUNet emerge as consistently strong performers. Therefore, for comprehensive segmentation of lychee images, these two models represent the most effective choices.

However, the segmentation performance of the endocarp and epicarp is relatively poor, with the MIoU, Dice, Recall, and Precision indices mostly below 0.80. This is likely due to the complex and relatively small structure of these two components, compared to the whole lychee fruit and pulp, and deep learning models may omit some of the information in order to improve generalization. The pericarp of mature lychee is approximately 400-500 *µm* thick and consists of three layers: the epicarp, the mesocarp and the endocarp (Riederer et al., 2015). Further studies could consider using multi-scale or pyramid-based architectures to specifically improve segmentation of the endocarp and the epicarp.

### 3.3 Efficacy of trait extraction

**Figure 6:**
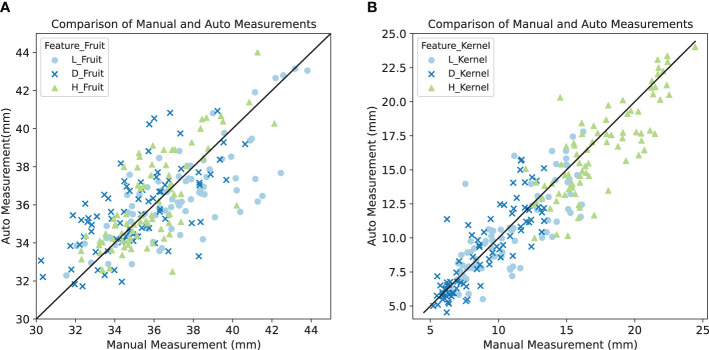
Comparison between manual and auto measurements for **(A)** fruit features and **(B)** kernel features. The x-axis represents the manual measurements of these morphological traits, which are considered the gold standard in our analysis. The y-axis displays the corresponding automatic measurements derived from our image segmentation algorithms. Each point on the scatter plots represents an individual measurement, with different colors symbolizing different traits: skin blue for length, dark blue for width, and green for height.

The study further explored the effectiveness of traits extraction by comparing them to the manual measurements. **Figure 6** presents this comparative analysis for the dimensions — length, width, and height — of both the kernel and the fruit. Specifically in **Table 2**, the MSE and RMSE values for parameter measurements are detailed, showcasing the performance metrics across different experimental conditions. In practical terms, for a lychee fruit with dimensions around 30mm, the automated measurement method incurs an error margin of approximately 1.5 mm. Similarly, for a lychee kernel measuring about 10mm, the error margin of the automated measurements is around 1.3 mm. These findings solidly affirm the reliability and precision of the automated measurement approach. The method is especially accurate in evaluating kernel dimensions, showcasing its effectiveness in detailed trait assessment.

**Table 2:** MSE and RMSE values for parameter measurements.

	L_Fruit	D_Fruit	H_Fruit		L_Kernel		D_Kernel	H_Kernel
Mean Absolute Error (MAE)	1.44	1.58	1.26		1.39		1.24	1.66
Root Mean Squared Error (RMSE)	1.89	1.98	1.61		1.94		1.61	2.16

### 3.4 Significance of traits for cultivar differentiation

**Figure 7:**
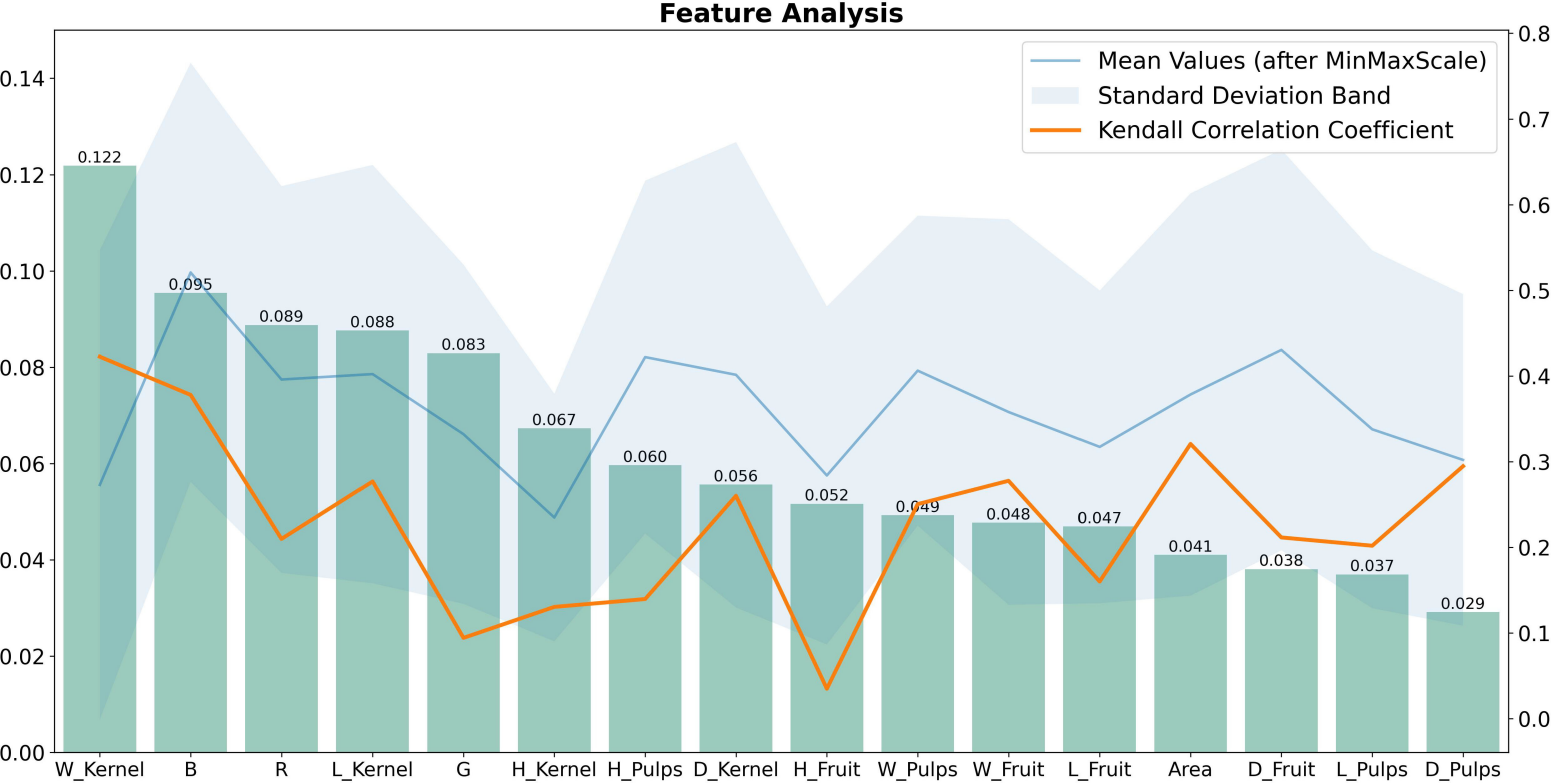
Feature analysis. The left y-axis of the figure shows a histogram ranking these traits based on their significance by a Random Forest Classifier. The right y-axis is for the two lines within a shaded area. The orange line charts the Kendall Correlation Coefficient to the species variables. The blue line represents the average value of each trait after applying MinMax scaling, surrounded by the blue standard deviation zone.

**Figure 8:**
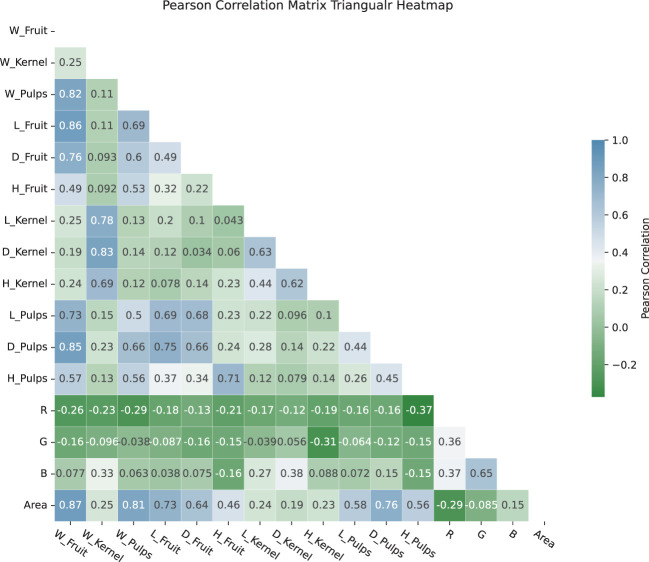
Pearson correlation matrix triangular heatmap. Each cell within the heatmap provides the correlation coefficient between two attributes, with the color intensity and direction (green to blue) indicating the strength and type of the relationship.

In the feature selection process, this study employed both the Random Forest method and the Kendall Coefficient method, as each method offers insights into feature importance. The Random Forest approach gives insight into how features contribute to the performance of a specific model, and the Kendall rank correlation coefficient provides a quantify strength of the monotonic relationship between variables (Lillo-Bravo et al., 2023). **Figure 7** is an exploration on the significance of lychee traits, which consists of two y-axis referring to the two methods. According to the left yaxis, the ‘W Kernel’ trait, with a value of 0.122, emerges as the most significant trait identified by the Random Forest Classifier, followed by traits such as ‘B’, ‘R’, ‘L Kernel’ and ‘G’, with respective values of 0.095, 0.089, 0.088, 0.083 in descending order. While right y-axis, employing the Kendall Correlation Coefficient method, also identifies ‘W Kernel’ as the most crucial trait. However, traits like ‘G’, ‘H Kernel’, ‘H Pulps’, ‘H Fruit’, ‘Area’ and ‘D Pulps’ exhibit an inverse pattern compared to the Random Forest method. This suggests that these traits may have complex relationship with the classification variables. Combined with the standard deviation band in the figure, the values of the individual features fluctuate widely, so that we believe that all features need to be taken into account ultimately. Moreover, **Figure 8** illustrates a Pearson Correlation Matrix Triangular Heatmap, which effectively showcases the interrelationships among different fruit attributes.

**Figure 9:**
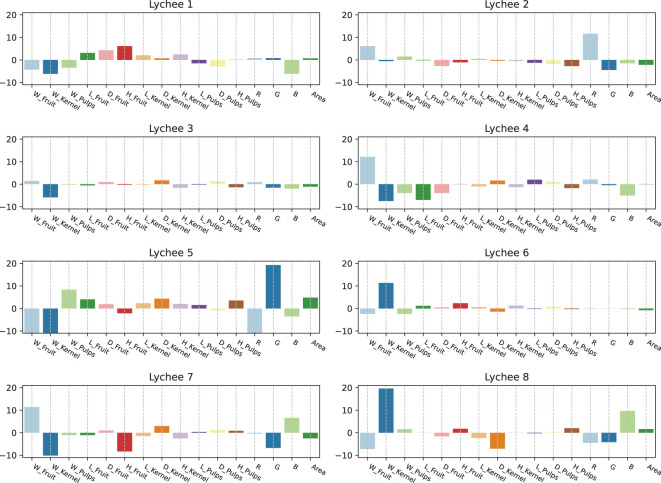
Importance of trait features in different lychee varieties. Each subplot represents a particular lychee variety, with the vertical axis indicating the level of importance for each trait, and the horizontal axis representing different feature types.

The dataset in this study includes eight unique lychee species, each defined by distinctive traits that set them apart. Linear Discriminant Analysis (LDA) was applied to ascertain the significance of each trait in differentiating between the lychee varieties. **Figure 9** provides a visual representation of the importance of various trait features across these eight lychee species. The data reveal pronounced differences in color and weight traits, and more subtle variations in size traits. This underscores the critical role of trait features in distinguishing among different varieties of lychee.

### 3.5 Classification accuracy with machine learning

Understanding the importance of phenotypic traits for each lychee species, further work was conducted to classify lychees using machine learning methods. Models would use the morphological traits extracted from lychee CT images listed in **Table 1** as input, and the species as output. Given the challenges posed by small datasets, selecting the appropriate classifier and cross-validation (CV) method is crucial to ensure robustness and accurate performance evaluation of the model. Three different classifiers were employed: Support Vector Machine (SVM), Random Forest Classifier (RFC), and Linear Discriminant Analysis (LDA).

Additionally, the models were trained using two distinct CV methods: Stratified Cross-Validation (Stratified CV) and Leave-One-Out Cross-Validation (LOO-CV), to extract the maximum information from a limited dataset by repeatedly splitting the dataset into multiple subsets for training and testing. In the Stratified CV method with *n splits* = 5 for a dataset, the dataset is divided into 5 folds, ensuring that in each iteration, the training and testing sets maintain proportional representation of all 8 species, with 4 folds used for training and 1 fold for testing. In LOO-CV method, the dataset is iteratively split into training and testing sets for each sample, with one sample left out for testing in each iteration while the remaining samples are used for training.

**Figure 10:**
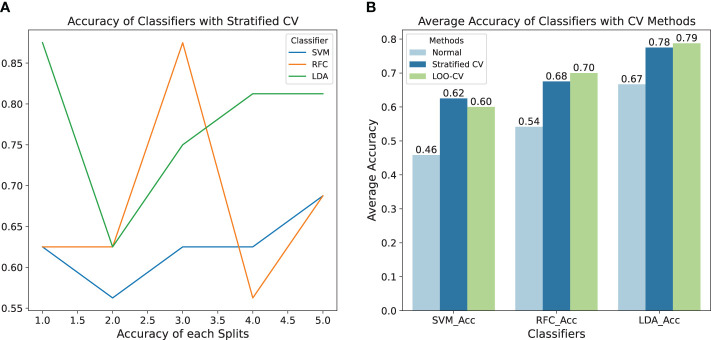
**(A)** Accuracy of classifiers with stratified CV method, which illustrates the accuracy of classifiers using the Stratified CV method across different splits. **(B)** Average accuracy of classifiers with and without CV methods, where the sky-blue bars indicate the accuracy of models trained without CV methods, serving as a baseline for comparison.

The left subfigure in **Figure 10** illustrates the accuracy of classifiers using the Stratified CV method across different splits. It is observed that the LDA algorithm outperforms the other two, demonstrating higher average accuracy and lower standard deviation, indicating its relative effectiveness in this application. The results are depicted on the right side. It is evident that models trained with CV methods surpass the baseline models in performance, with LOO-CV yielding the most favorable results. This superiority can be attributed to LOO-CV’s robustness and reduced susceptibility to overfitting, which are essential attributes for handling small datasets. Specifically, the LDA algorithm achieves an accuracy of 0.78 with the LOO-CV method and 0.79 with the Stratified-CV method, making it a reliable choice for such scenarios, which are highly recommended for classification tasks involving small sample sizes.

Attributed to the combination of a limited sample size and relatively high feature dimensions, the potential improvement in classification results could be achieved by increasing the size of the data sample, implementing dimensionality reduction, or exploring the application of a deep learning model. The sparsity of data in higher-dimensional spaces necessitates a larger dataset for effective modeling and generalization. Particularly with small samples, this scenario may result in models failing to adequately capture the overall data structure.

## 4 Discussion

### 4.1 Advantages and limitations of photon-counting micro-CT

The incorporation of a photon-counting micro-CT system in our study has significantly enhanced the resolution and detail in the imaging of lychee fruits. This advanced technology, known for its high resolution and multi-energy spectrum analysis, has allowed us to observe intricate details within the lychee fruits, which are critical for accurate phenotypic trait extraction. These features enable it to discover more details that conventional CT systems might miss. The ability to analyze multiple energy spectrums allows for a more nuanced and detailed understanding of the scanned subject, which is particularly beneficial in complex biological studies. While the photon-counting micro-CT system offers many advantages, there are certain challenges and limitations to consider. Its specialization in small-scale imaging may limit its applicability in larger subjects or broader clinical settings. Additionally, the complexity and cost of the technology may pose challenges for widespread adoption in varied research environments.

### 4.2 Broader implications for scientific research

The photon-counting micro-CT system, as demonstrated in our study, holds significant potential for advancing plant phenomics research. Its ability for material decomposition, exemplified by the quantification of sucrose (Kanno and Kuroyama, 2021), organic acids, vitamins and important minerals compounds, introduces an innovative dimension in plant biology for the analysis of internal compositions and phenotypic attributes. It could also transform the way we understand root systems (Mooney et al., 2012), seed germination, and plant-microorganism interactions, providing insights into plant growth, disease resistance, and nutrient uptake. The system’s detailed imaging capabilities allow for non-destructive analysis, making it an invaluable tool for both plant scientists and agriculturalists in exploring and understanding the intricate details of plant life at a micro-level.

## 5 Conclusions

This study has for the first time utilized photon-counting micro-CT system to analyze phenotypic traits in lychee cultivars, as well as the plants phenotypes, marking a significant advance in plant phenomics. The approach integrates high-resolution imaging with advanced deep learning models, enabling precise segmentation and analysis of lychee fruit structures. This methodology not only demonstrates high accuracy in distinguishing different cultivars but also sets a new standard in non-destructive plant trait analysis. By combining detailed imaging with robust data processing techniques, our study opens up new possibilities for research in plant biology and agricultural sciences, offering a novel and efficient tool for cultivar classification and in-depth trait examination.

## Data availability statement

The original contributions presented in the study are included in the article/supplementary material. Further inquiries can be directed to the corresponding author.

## Author contributions

MX: Writing – review & editing, Data curation, Investigation. SH: Conceptualization, Visualization, Writing – original draft. WX: Methodology, Resources, Writing – review & editing. TX: Funding acquisition, Project administration, Resources, Supervision, Writing – review & editing.
